# High-resolution intracranial vessel wall MRI in an elderly asymptomatic population: comparison of 3T and 7T

**DOI:** 10.1007/s00330-016-4483-3

**Published:** 2016-07-07

**Authors:** Anita A. Harteveld, Anja G. van der Kolk, H. Bart van der Worp, Nikki Dieleman, Jeroen C. W. Siero, Hugo J. Kuijf, Catharina J. M. Frijns, Peter R. Luijten, Jaco J. M. Zwanenburg, Jeroen Hendrikse

**Affiliations:** 1 Department of Radiology, University Medical Center Utrecht, Postbox 85500, 3508 GA Utrecht, The Netherlands; 2 Department of Neurology and Neurosurgery, Brain Center Rudolf Magnus, University Medical Center Utrecht, Utrecht, The Netherlands; 3 Image Sciences Institute, University Medical Center Utrecht, Utrecht, The Netherlands

**Keywords:** Intracranial atherosclerosis, Magnetic resonance imaging, Cerebral arteries, Cerebrovascular disorders, Neuroimaging

## Abstract

**Objectives:**

Several intracranial vessel wall sequences have been described in recent literature, with either 3-T or 7-T magnetic resonance imaging (MRI). In the current study, we compared 3-T and 7-T MRI in visualising both the intracranial arterial vessel wall and vessel wall lesions.

**Methods:**

Twenty-one elderly asymptomatic volunteers were scanned by 3-T and 7-T MRI with an intracranial vessel wall sequence, both before and after contrast administration. Two raters scored image quality, and presence and characteristics of vessel wall lesions.

**Results:**

Vessel wall visibility was equal or significantly better at 7 T for the studied arterial segments, even though there were more artefacts hampering assessment. The better visualisation of the vessel wall at 7 T was most prominent in the proximal anterior cerebral circulation and the posterior cerebral artery. In the studied elderly asymptomatic population, 48 vessel-wall lesions were identified at 3 T, of which 7 showed enhancement. At 7 T, 79 lesions were identified, of which 29 showed enhancement. Seventy-one percent of all 3-T lesions and 59 % of all 7-T lesions were also seen at the other field strength.

**Conclusions:**

Despite the large variability in detected lesions at both field strengths, we believe 7-T MRI has the highest potential to identify the total burden of intracranial vessel wall lesions.

***Key Points*:**

• *Intracranial vessel wall visibility was equal or significantly better at 7-T MRI*

• *Most vessel wall lesions in the cerebral arteries were found at 7-T MRI*

• *Many intracranial vessel wall lesions showed enhancement after contrast administration*

• *Large variability in detected intracranial vessel wall lesions at both field strengths*

• *Seven-tesla MRI has the highest potential to identify total burden of intracranial atherosclerosis*

**Electronic supplementary material:**

The online version of this article (doi:10.1007/s00330-016-4483-3) contains supplementary material, which is available to authorized users.

## Introduction

Intracranial atherosclerosis is one of the main causes of ischaemic stroke and transient ischaemic attack (TIA), and has been associated with a higher risk of recurrent stroke [1, 2]. Current standard non-invasive imaging methods, like computed tomography (CT) angiography, visualise the lumen of the intracranial vasculature instead of its vessel wall, thereby risking underestimation of intracranial atherosclerosis in ischaemic stroke [3–6] due to compensatory arterial remodelling that maintains lumen diameter [4, 7]. Therefore, several magnetic resonance imaging (MRI) sequences have recently been developed for direct evaluation of the intracranial vessel wall and its pathology in vivo [8]. Studies have shown that high-resolution MRI is able to identify intracranial vessel wall abnormalities even before causing luminal narrowing.

Intracranial vessel wall imaging has been performed mainly at 3.0- and 7.0-tesla (T) field strengths. At these higher magnetic field strengths, the increased signal-to-noise (SNR) and contrast-to-noise ratios (CNR) can be exploited for imaging at a higher spatial resolution within reasonable scan times to clearly show the thin arterial vessel walls, and for improvement of lesion conspicuousness [7, 9]. Current challenges at 3 T are incomplete cerebrospinal fluid (CSF) suppression, potentially limiting vessel wall assessment, and (often) limited coverage [8, 10, 11]. Seven-tesla MRI offers the advantage of an increased SNR, allowing for complete CSF suppression and whole-brain imaging within clinically feasible scan times [12]; its use, however, is currently hampered by restricted availability and increased transmit field (B_1_^+^) inhomogeneity, causing artefacts that can limit vessel wall assessment [13]. The implications of these differences are unclear, because a direct comparison between intracranial vessel wall MRI at these field strengths has not yet been performed. The purpose of this study was to compare visualisation of the intracranial vessel wall and possible vessel wall lesions between 3-T and 7-T MRI.

## Methods

### Study population

This prospective study was approved by the institutional review board of our institution; all subjects provided written informed consent. Between November 2013 and December 2014, volunteers aged >50 years, without a history of cerebrovascular or ischaemic heart disease or contraindications for MR imaging, were included. These volunteers formed the control group of the ongoing PIVI study (Posterior Intracranial Vessel wall Imaging; NTR5688, www.trialregister.nl).

### Imaging

All subjects underwent MRI at both 3 T and 7 T. For each field strength, an optimised, T_1_-weighted intracranial vessel wall imaging sequence was used; a previous ex vivo study at 7 T [14] showed T_1_-weighted imaging to have the most promising image contrast for visualising and characterising intracranial arterial vessel wall lesions. In addition, a T_1_-weighted sequence enables assessment of vessel-wall contrast enhancement. The sequence at 3 T had been optimised in volunteers (data not shown) based on a previously published sequence by Qiao et al. [10] The 7-T sequence had already been optimised in previous studies [12, 15]. At both field strengths, the intracranial vessel wall scan was performed before and after contrast administration; a minimum of 12 h was taken between both MRI examinations, to make sure the contrast agent had washed out sufficiently, and care was taken that both MRI examinations were planned as close after each other as possible (median, 4 days; IQR, 3-9 days). The postcontrast vessel wall scan was acquired approximately 5 min after intravenous administration of 0.1 mL/kg of a gadolinium-containing contrast agent (Gadobutrol, Gadovist 1.0 mmol/mL; Bayer Schering Pharma, Newbury, UK). For signal improvement in the cerebellar region at 7 T, high permittivity dielectric pads were used (Leiden University Medical Center, Leiden, The Netherlands) [16, 17].

#### 3-T MRI protocol

Imaging was performed on a 3-T whole-body system (Achieva; Philips Healthcare, Best, The Netherlands), with a quadrature body coil for transmission and an eight-channel head coil for reception. The imaging protocol included a three-dimensional (3D) T_1_-weighted volumetric isotropically reconstructed turbo spin-echo acquisition (VIRTA) intracranial vessel wall sequence (adapted from Qiao et al. [10] by Dieleman et al., submitted for publication). The applied scan parameters are presented in Table 1.

**Table 1:** Scan parameters of the intracranial vessel wall imaging sequence at 3 T and 7 T

Scan parameter	3-T VIRTA^a^	7-T MPIR-TSE^b^
FOV (mm^3^)	200 × 167 × 45	250 × 250 × 190
Acquisition orientation	Transverse oblique	Sagittal
Acquired spatial resolution (mm^3^)	0.6 × 0.6 × 1.0	0.8 × 0.8 × 0.8
Reconstructed spatial resolution (mm^3^)	0.5 × 0.5 × 0.5	0.49 × 0.49 × 0.49
TR / TE / TI (ms)	1,500 / 36 / -	3,952 / 37 / 1,375
Flip angle (degrees)	90	120
Echo spacing (ms)	4.0	3.3
TSE factor	62 (incl. 6 start-up)	169 (incl. 10 start-up)
Oversampling factor	1.8	1
NSA	1	2
SENSE factor	1.5 (RL)	2 (AP) & 3 (RL)
Acquisition time (min:s)	6:51	10:40

*FOV* field-of-view, *MPIR-TSE* magnetisation-prepared inversion recovery, *NSA* number of signal averages, *SENSE* sensitivity encoding, *TE* echo time, *TI* inversion time, *TR* repetition time, *TSE* turbo spin echo, *VIRTA* volumetric isotropically reconstructed turbo spin-echo acquisition

^a^Sequence was planned so that as much of the circle of Willis as possible was within the FOV. Several parameters were adapted/added to Qiao et al. [10]: TR = 1,500 ms and anti-DRIVen Equilibrium (DRIVE) module to increase T_1_-weighting and CSF suppression; a low minimum flip angle (25 degrees) in the variable flip angle refocusing pulse train for increased flow suppression [18]; interpolation factor = 2 by zero-padding in the slice direction during reconstruction, slight adjustment of the acquired in-plane resolution, and reducing the TR to reduce scan time further

^b^In comparison to previous studies [12, 15, 19], sequences were obtained with a dual transmit system that provides a B_1_^+^ in the brain that matches the nominal flip angle; therefore, all flip angles could be reduced by 20 % to obtain the same image contrast

#### 7-T MRI protocol

For 7-T MRI, a whole-body system (Philips Healthcare, Cleveland, OH, USA) was used with a 32-channel receive coil and volume transmit/receive coil for transmission (Nova Medical, Wilmington, MA, USA). The imaging protocol included a 3D whole-brain T_1_-weighted magnetisation-prepared inversion recovery turbo spin echo (MPIR-TSE) intracranial vessel wall sequence [15]. The applied scan parameters are presented in Table 1.

### Image assessment

Images were assessed in the same format as they are normally evaluated in clinical practice, which includes the ‘standard’ image interpolation performed by the scanner software. Multiplanar reconstructions (MPRs) were made from the vessel-wall scans acquired at 3 T (thickness, 1.0 mm; no slice gap) and 7 T (thickness, 0.8 mm; no slice gap), using a standalone workstation (Philips). The used slice thickness was based on the acquired resolution in the slice direction. To obtain the same data sets for further analysis, the 7-T vessel-wall MPRs were made using the spatial orientation of the 3-T images.

Three-tesla and 7-T MPR images were evaluated in random order by two trained raters (A.K. and A.H.), with 6 and 3 years of experience in assessing intracranial vessel wall images, respectively, who were blinded for each other’s assessment and for the findings on the other (3-T or 7-T) scan. Each scan was analysed once by each rater; in case of disagreement regarding vessel wall lesions, a consensus reading was performed with a third rater (J.H.; 6 years of experience in assessing intracranial vessel wall images). After consensus, non-corresponding vessel-wall lesions at 3 T and 7 T were assessed in a side-by-side comparison.

The assessed vessel segments included: anterior cerebral artery (ACA; A1 and A2 segments); anterior communicating artery (ACoA); middle cerebral artery (MCA; M1, M2 and M3 segments); internal carotid artery (ICA; distal intracranial segment; intracranial bifurcation); posterior communicating artery (PCoA); posterior cerebral artery (PCA; P1 segment, bifurcation P1-P2 and P2 segment); basilar artery (BA; proximal half, distal half and bifurcation with P1 segment); vertebral arteries (VA; proximal and distal half).

#### Image quality

Image quality was evaluated using the method modified from Van der Kolk et al. [15], with three qualitative grading scales for overall artefacts (0 = artefacts hampering assessment; 1 = moderate artefacts, but images assessable; 2 = no artefacts), overall visibility of the arterial vessel wall (0 = poor; 1 = moderate; 2 = good), and visibility of all separate arterial vessel walls (0 = outside FOV; 1 = not visible; 2 = poor; 3 = moderate; 4 = good).

#### Vessel-wall lesions

Vessel-wall lesions were scored on the precontrast images according to the methods previously described [12, 20] A vessel-wall lesion was defined as either a clear focal or more diffuse thickening of the vessel wall, compared with the healthy contralateral or neighbouring vessel wall [21]. For assessment of contrast enhancement, vessel wall images were processed using MeVisLab (version 2.7; MeVis Medical Solutions, Bremen, Germany). After coregistering the postcontrast scan to the precontrast scan using elastix [22], the precontrast scan was subtracted from the (coregistered) postcontrast scan. The resulting subtracted images together with the precontrast and postcontrast vessel wall images were used for contrast enhancement assessment [12]. In addition to contrast enhancement, specific lesion characteristics (configuration and thickening pattern) were also assessed [*see* Electronic supplementary material (ESM)].

### Statistical analysis

Differences between image quality ratings at 3 T and 7 T were calculated using a non-parametric Wilcoxon signed-rank test. Inter-rater agreement of the number and location of vessel wall lesions was evaluated using the Dice’s similarity coefficient (DSC) [23] for the 3-T and 7-T MR images separately. For inter-rater agreement of contrast enhancement assessment Cohen’s kappa was calculated. Statistical analyses were performed using IBM SPSS Statistics (version 21; IBM Corporation, Armonk, NY, USA). A *p* value of <0.05 was considered to be statistically significant. Bonferroni correction for multiple comparisons was applied when appropriate.

## Results

### Study population

Twenty-one healthy volunteers (12 men; age 66 ± 5 years) were included. Baseline characteristics are shown in ESM Table I.

### Image quality

Since image quality ratings of both raters were comparable, the mean of both raters was used. Seven-tesla images showed significantly more artefacts compared with 3 T, both precontrast and postcontrast (Table 2). For analysis of vessel wall visibility, subjects scored with artefacts hampering assessment on either 3-T or 7-T images where the majority of the vessel walls were affected (i.e. due to severe motion artefacts) were excluded (*n* = 6; motion artefacts at 3 T (*n* = 2) or 7 T (*n* = 4); precontrast *n* = 2 and postcontrast *n* = 4). Overall vessel wall visibility was scored significantly better for the 7-T images compared with 3 T, both precontrast and postcontrast (Table 2). On arterial segment level (Table 3), the ICA, ACA and proximal MCA (M1 segment) vessel walls were significantly better visible at 7 T; this was also the case for the P2 segment.

**Table 2:** Qualitative scoring of artefacts and overall visibility of the arterial vessel wall on 3-T and 7-T MRI (precontrast and postcontrast)

	Precontrast			Postcontrast		
	3 T	7 T	*p*-value^c^	3 T	7 T	*p-value* ^*c*^
Artefacts^a^						
0	2 (5 %)	14 (33 %)		6 (14 %)	16 (38 %)	
1	29 (69 %)	28 (67 %)		32 (76 %)	26 (62 %)	
2	11 (26 %)	0 (0 %)		4 (10 %)	0 (0 %)	
Proportion of overall agreement (%)	76	62		81	71	
Mean rater 1 (range)	1.24 (0-2)	0.57 (0-1)		0.95 (0-2)	0.57 (0-1)	
Mean rater 2 (range)	1.19 (0-2)	0.76 (0-1)		0.95 (0-2)	0.67 (0-1)	
Mean both raters	1.21	0.67	<0.001	0.95	0.62	0.024
Overall visibility vessel wall^b^						
0	5 (17 %)	3 (10 %)		5 (17 %)	3 (10 %)	
1	22 (73 %)	11 (37 %)		23 (77 %)	14 (47 %)	
2	3 (10 %)	16 (53 %)		2 (7 %)	13 (43 %)	
Proportion of overall agreement (%)	60	80		80	87	
Mean rater 1 (range)	0.87 (0-2)	1.40 (0-2)		0.93 (0-2)	1.33 (0-2)	
Mean rater 2 (range)	1.00 (0-2)	1.47 (0-2)		0.87 (0-2)	1.33 (0-2)	
Mean both raters	0.93	1.43	0.009	0.90	1.33	0.019

Grading scale overall artefacts: 0 = artefacts hampering assessment; 1 = moderate artefacts, but images assessable; 2 = no artefacts

Grading scale overall visibility of the arterial vessel wall: 0 = poor; 1 = moderate; 2 = good

^a^Based on 21 subjects

^b^Based on 15 subjects

^c^Bonferroni corrected significance level *p* < 0.025 (corrected for two comparisons of rating scales)

**Table 3:** Qualitative visibility scoring of all separate arterial vessel wall segments of the circle of Willis and its primary branches on 3-T and 7-T MRI (precontrast)^a^

Location	3 T	7 T	*p* - value^b^
Anterior cerebral artery			
A1 segment	2.21 (1-3)	2.79 (2-4)	0.001*
A2 segment	1.63 (1-3)	2.24 (1-4)	<0.001*
Anterior communicating artery	1.16 (1-2)	1.32 (1-3)	0.398
Middle cerebral artery			
M1 segment	2.11 (1-3)	2.76 (2-4)	<0.001*
M2 segment	2.16 (1-3)	2.24 (1-3)	0.802
M3 segment	1.34 (0-3)	1.05 (0-2)	0.052^c^
Internal carotid artery			
Distal intracranial segment	2.82 (2-3)	3.37 (2-4)	0.002*
Intracranial bifurcation	2.61 (2-4)	3.16 (2-4)	<0.001*
Posterior communicating artery	1.90 (1-3)	2.50 (1-4)	0.083
Posterior cerebral artery			
P1 segment	2.55 (1-4)	3.13 (2-4)	0.003
Bifurcation	2.26 (1-4)	3.08 (2-4)	0.004
P2 segment	1.79 (1-3)	2.63 (2-3)	<0.001*
Basilar artery			
Bifurcation	2.74 (1-4)	2.90 (1-4)	0.413
Distal half	2.97 (2-4)	2.90 (1-4)	0.499
Proximal half	3.11 (1-4)	2.97 (1-4)	0.545
Vertebral artery			
Distal half	3.26 (2-4)	2.84 (1-4)	0.168
Proximal half	3.32 (2-4)	2.97 (1-4)	0.214

*statistically significant (after Bonferroni correction)

Scores are given as mean (range) of both raters

Grading scale: 0 = outside FOV; 1 = not visible; 2 = poor; 3 = moderate; 4 = good

^a^Based on 19 subjects per location

^b^Bonferroni corrected significance level *p* < 0.003 (corrected for 17 comparisons of arterial segments)

^c^Rating “0” was excluded from statistical analysis (*n* = 3 subjects)

### Vessel-wall lesions

For analysis of vessel-wall lesions, subjects with poor overall vessel wall visibility scored by both raters on either 3-T or 7-T precontrast images were excluded [*n* = 5; poor visibility at 3 T only (*n* = 1), 7 T only (*n* = 2), or both at 3 T and 7 T (*n* = 2)], resulting in 16 subjects for analysis. For analysis of lesion enhancement, an additional three subjects were excluded based on poor overall visibility of the postcontrast scan [poor visibility at 3 T only (*n* = 2) or 7 T only (*n* = 1)].

Pre-consensus inter-rater agreement for number and location of the identified vessel-wall lesions was good at both 3 T (DSC, 0.68) and 7 T (DSC, 0.67). Also, a good to excellent inter-rater agreement was found for lesion contrast enhancement (kappa 0.85 for 3 T, and 0.67 for 7 T). On the precontrast scans, 45 (at 3 T) and 67 (at 7 T) vessel-wall lesions were scored after consensus; an additional 3 (at 3 T) and 12 (at 7 T) lesions were identified because of vessel-wall enhancement. In total, 48 vessel-wall lesions were identified at 3 T (mean, 3 per subject; range, 1-7), of which 7 (15 %) showed enhancement (Table 4). At 7 T, 79 lesions were identified (mean, 5 per subject; range, 1-10), of which 29 (37 %) showed enhancement (Table 4).

**Table 4:** Overview of number, location and enhancement of identified vessel wall lesions on 3-T and 7-T vessel wall images, as well as lesions that corresponded between 3 T and 7 T

	3 T		7 T		Corresponding 3 T-7 T		
	Lesions	Enhancement^a^	Lesions	Enhancement^b^	Lesions	Enhancement^c^	
Location						3 T	7 T
Total anterior circulation	17 (35.4)	1	32 (40.5)	8	7	1	4
Anterior cerebral artery	3 (6.3)	0	3 (3.8)	0	0	0	0
A1 segment	2 (4.2)	0	1 (1.3)	0	0	0	0
A2 segment	1 (2.1)	0	2 (2.5)	0	0	0	0
Anterior communicating artery	0 (0.0)	0	0 (0.0)	0	0	0	0
Middle cerebral artery	6 (12.5)	1	13 (16.5)	2	3	1	2
M1 segment	5 (10.4)	1	9 (11.4)	2	3	1	2
M2 segment	1 (2.1)	0	4 (5.1)	0	0	0	0
M3 segment	0 (0.0)	0	0 (0.0)	0	0	0	0
Internal carotid artery	8 (16.7)	0	16 (20.3)	6	4	0	2
Distal intracranial segment	3 (6.3)	0	6 (7.6)	3	1	0	1
Intracranial bifurcation	5 (10.4)	0	10 (12.7)	3	3	0	1
Total posterior circulation	31 (64.6)	6	47 (59.5)	21	17	6	12
Posterior communicating artery	2 (4.2)	0	2 (2.5)	0	1	0	0
Posterior cerebral artery	4 (8.3)	0	11 (13.9)	1	1	0	0
P1 segment	2 (4.2)	0	0 (0.0)	0	0	0	0
Bifurcation	0 (0.0)	0	4 (5.1)	0	0	0	0
P2 segment	2 (4.2)	0	7 (8.9)	1	1	0	0
Basilar artery	8 (16.7)	0	10 (12.7)	3	4	0	2
Bifurcation	5 (10.4)	0	5 (6.3)	1	2	0	1
Distal half	3 (6.3)	0	2 (2.5)	1	2	0	1
Proximal half	0 (0.0)	0	3 (3.8)	1	0	0	0
Vertebral artery	17 (35.4)	6	24 (30.4)	17	11	6	10
Distal half	3 (6.3)	0	6 (7.6)	3	1	0	1
Proximal half	14 (29.2)	6	18 (22.8)	14	10	6	9
Total	48	7	79	29	24	7	16

Number of lesions per location (% from total)

^a^ *n* = 7 lesions not assessable on postcontrast scans

^b^ *n* = 13 lesions not assessable on postcontrast scans

^c^ *n* = 3 lesions not assessable on postcontrast scans

#### Side-by-side comparison

**Fig. 1:**
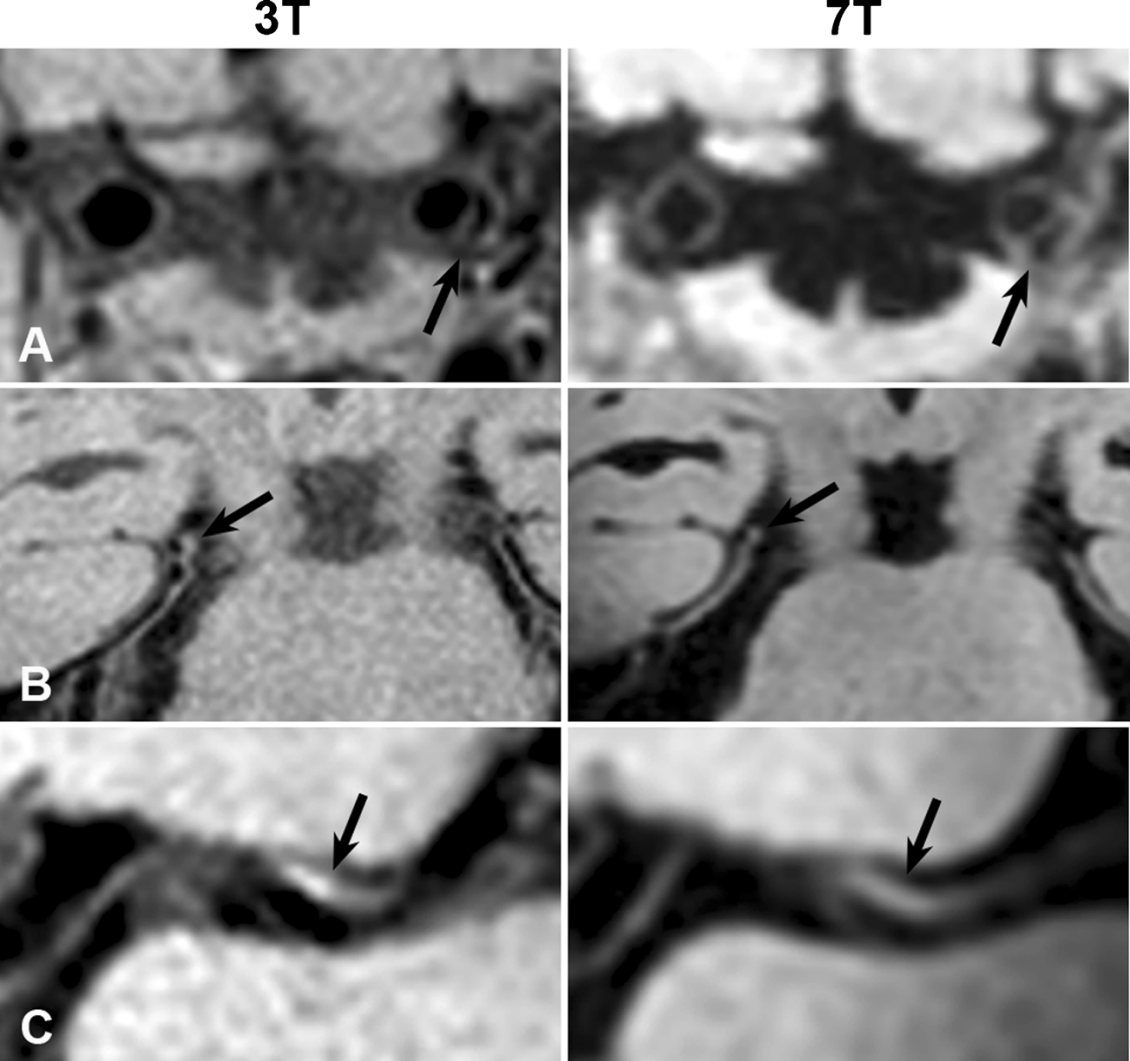
Corresponding vessel-wall lesions at 3 T and 7 T (*arrows*) on the precontrast images: located at the left distal intracranial segment of the ICA (**a**), right P2 segment of the PCA (**b**) and left M1 segment of the MCA (**c**). *ICA* intracranial internal carotid artery, *MCA* middle cerebral artery, *PCA* posterior cerebral artery

**Fig. 2:**
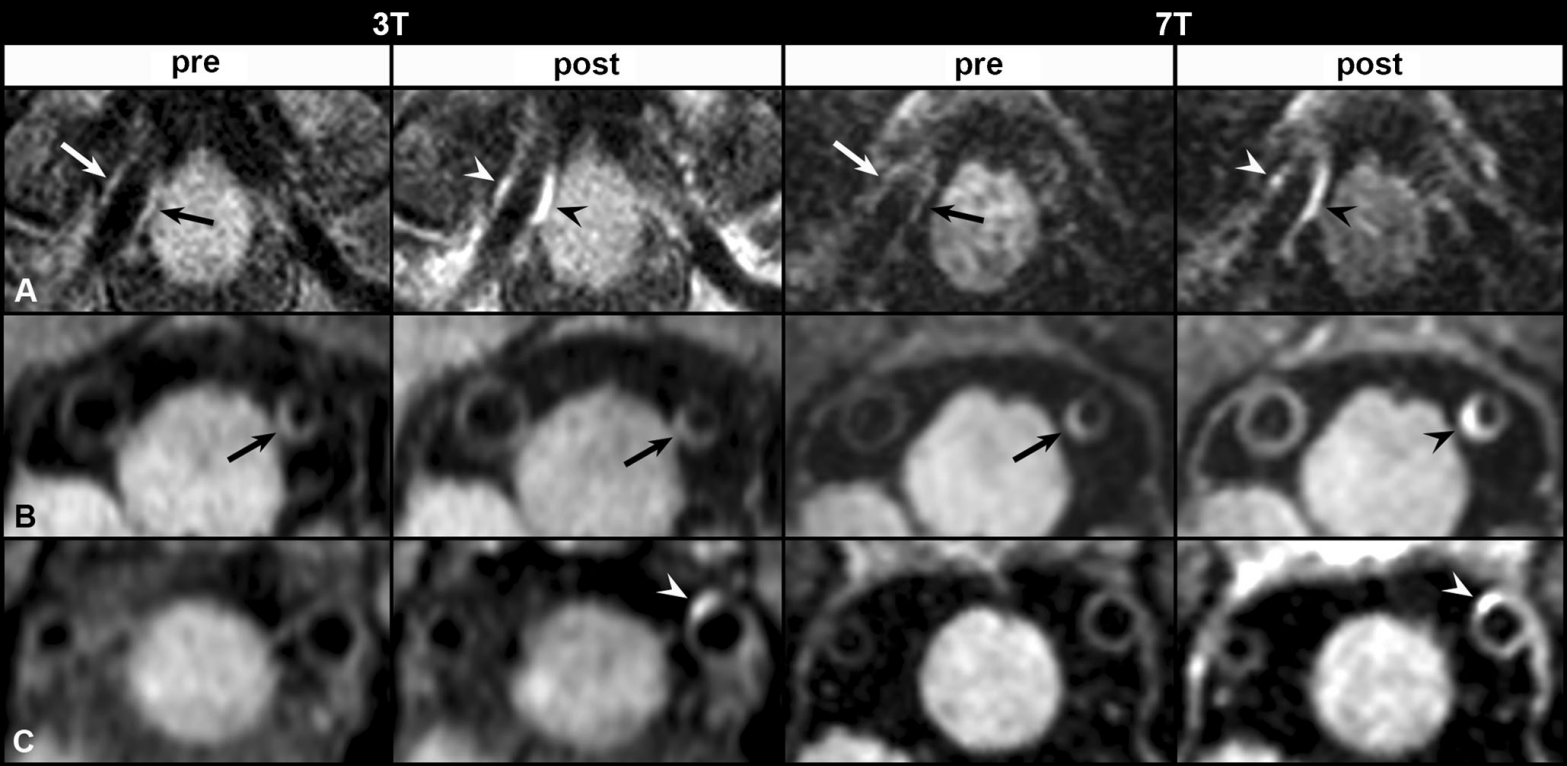
Vessel-wall enhancement after contrast administration. **a**, **b** Vessel-wall lesions identified on the precontrast 3 T and 7 T vessel wall images (*arrows*), with enhancement (*arrowheads*) on the postcontrast images at 3 T and 7 T (**a** right proximal VA), and 7 T only (**b** left proximal VA). **c** Vessel wall lesion identified based on enhancement of the vessel wall on the postcontrast 3 T and 7 T vessel wall images (**c** left proximal VA). *VA* vertebral artery

Twenty-four vessel wall lesions were identified at both 3 T and 7 T (Figs. 1 and 2). Almost one-third (7/24; 29 %) of lesions visible at both 3 T and 7 T showed enhancement on both field strengths; an additional one-third (9/24; 38 %) showed enhancement at 7 T only. Most corresponding lesions were present in the vertebral arteries, basilar artery and internal carotid arteries.

**Fig. 3:**
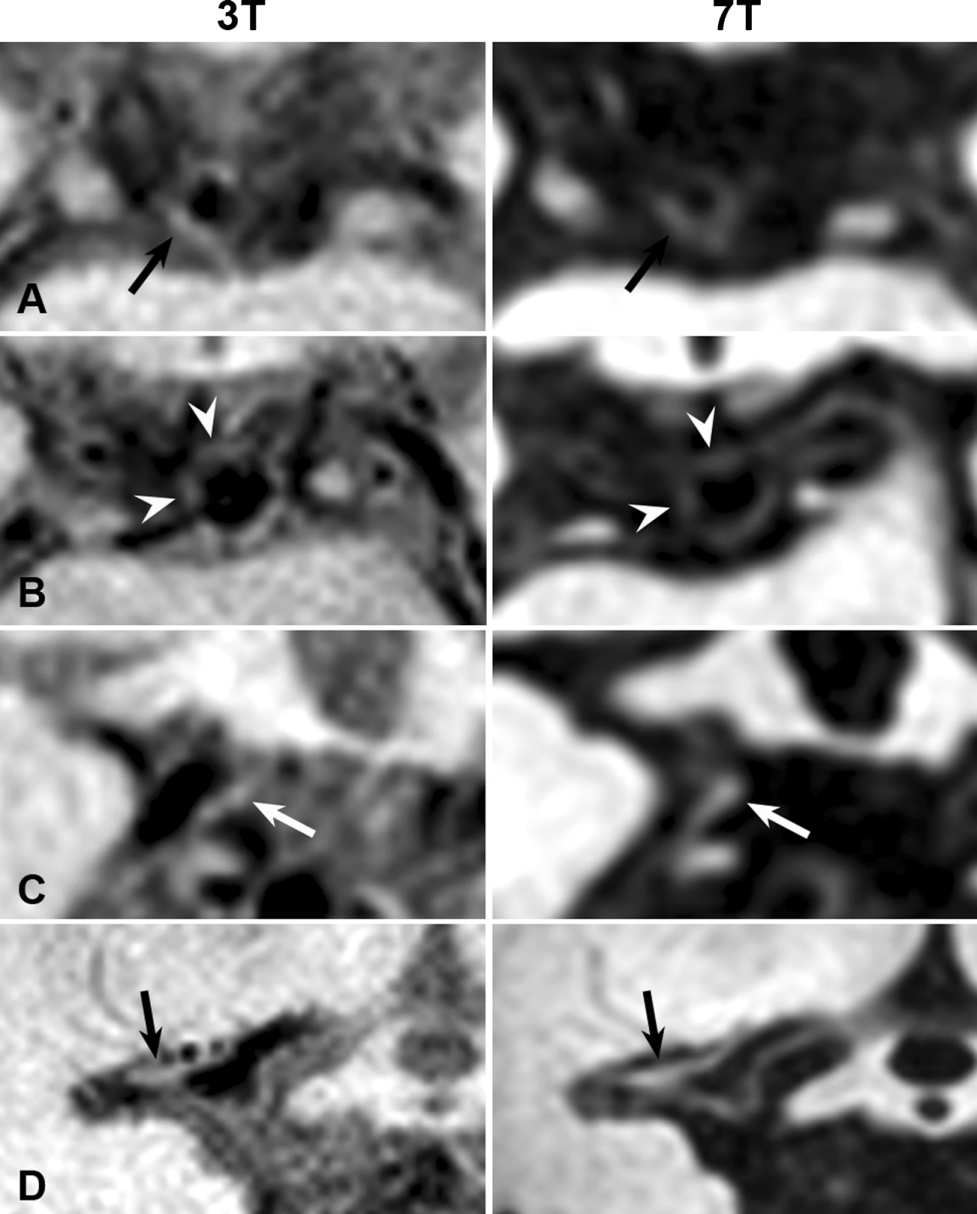
Non-corresponding intracranial vessel wall lesions (*arrows* or *arrowheads*) identified on either the 3-T (**a**, **b**) or 7-T (**c**, **d**) vessel wall images that were identified retrospectively on the other scan: located at the right P1 segment of the PCA (**a**), bifurcation BA-P1 (**b**), right bifurcation P1-P2 segment of the PCA (**c**), and right M1 segment of the MCA (**d**). *BA* basilar artery, *MCA* middle cerebral artery, *PCA* posterior cerebral artery

**Fig. 4:**
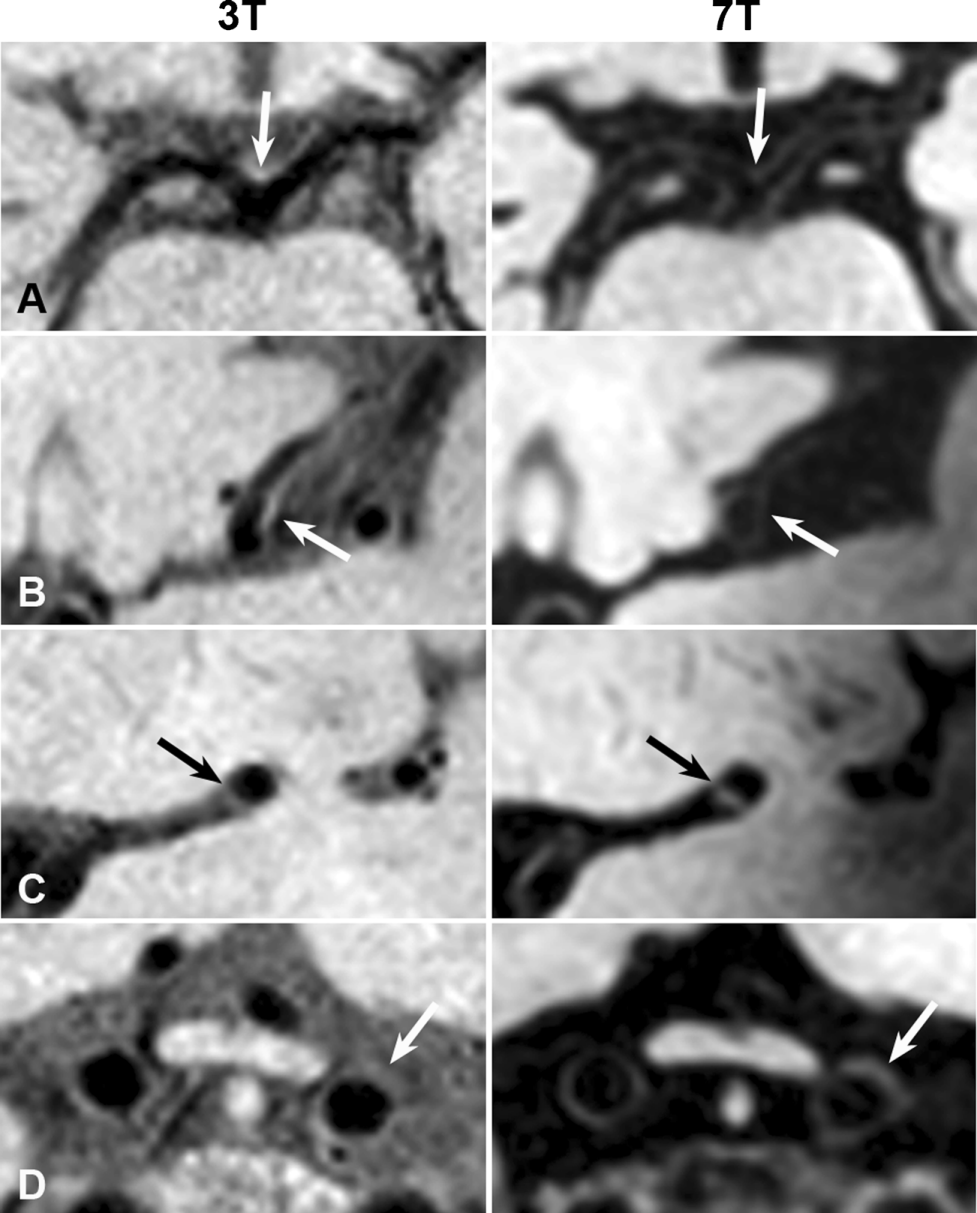
Non-corresponding intracranial vessel-wall lesions (*arrows*) identified on either the 3-T (**a**, **b**) or 7-T (**c**, **d**) vessel-wall images that could also not be identified on the other field strength retrospectively: located at the bifurcation BA-P1 (**a** visible at 3 T), left M2 segment of the MCA (**b** visible at 3 T), left M1 segment of the MCA (**c** visible at 7 T) and left distal intracranial segment of the ICA (**d** visible at 7 T). *BA* basilar artery, *ICA* internal carotid artery, *MCA* middle cerebral artery

Side-by-side comparison of the non-corresponding vessel-wall lesions showed that in retrospect, 10 lesions (21 %) scored only at 3 T and 23 lesions (29 %) scored only at 7 T were either visible retrospectively but missed at the other field strength by both raters, or identified but omitted during the consensus reading (Fig. 3). Fourteen (29 %) vessel-wall lesions that were identified at 3 T could not be retrospectively identified at 7 T. At 7 T, 32 (41 %) vessel wall lesions could not be retrospectively identified at 3 T (Fig. 4). The non-corresponding lesions were mainly located in areas where the vessel wall was less well visible; also, several of these lesions appeared smaller compared with the corresponding lesions. In total, 34 (71 %) of all lesions identified at 3 T (24 after consensus + 10 in retrospect) and 47 (59 %) at 7 T (24 after consensus + 23 in retrospect) were also seen on the other field strength.

## Discussion

In the current study, vessel wall visibility was equal or significantly better at 7 T compared with 3 T for the studied arterial segments, even though there were more artefacts hampering assessment at 7 T. Furthermore, more vessel wall lesions were scored on 7-T images. However, surprisingly, only 71 % of all 3-T lesions were seen at 7 T.

Better visualisation of the vessel wall at 7 T was most prominent in the proximal anterior cerebral circulation and the P2 segment of the PCA; the basilar artery and vertebral arteries were visualised equally good at both field strengths. The latter could be explained by better CSF suppression in this region compared to other brain regions at 3 T, probably because of higher local CSF flow pulsation. Artefacts at 7 T were mainly caused by motion artefacts or signal loss and B_1_^+^ inhomogeneities in the areas of the temporal lobes and cerebellum. Although motion artefacts were also present at 3 T, they occurred more frequently at 7 T. This might be related to the longer acquisition time of the 7-T sequence compared with 3 T, increasing the possibility of subject motion during scanning [24]. B_1_^+^ inhomogeneities at 7 T result in a spatially varying SNR and contrast that is most pronounced at the temporal lobes and cerebellum [13, 16]. In this study, dielectric bags were placed in the upper neck region of the subjects to improve imaging of the cerebellar region, which improved part of the signal loss, but did not reduce signal loss in all subjects.

Most vessel wall lesions were found using 7 T, which was expected since CSF suppression is better at 7 T than at 3 T in most brain regions. At 3 T, no explicit CSF suppression can be performed; therefore, suppression depends on the amount of CSF flow during the spin echo train which is spatially dependent, resulting in varying quality of CSF suppression in different brain regions. The 7-T sequence contains a non-selective adiabatic inversion pulse for global CSF suppression. Most corresponding lesions between 3 T and 7 T were present in the vertebral arteries, basilar artery, and internal carotid arteries: all larger arterial segments of the circle of Willis around which considerable CSF flow pulsation is present. These segments scored relatively good for vessel wall visibility at both 3 T and 7 T.

Close to one-half of 7-T lesions (41 %) could not be identified at 3-T MRI. On the other hand, a striking one-third (29 %) of all lesions identified at 3 T were not visible at 7 T. The latter might be explained by the higher acquired in-plane spatial resolution of the 3-T sequence (0.6 × 0.6 mm^2^) compared with the 7-T sequence (0.8 × 0.8 mm^2^), so that very small lesions seen at 3 T might have been missed at 7 T. The first could be explained by the increased SNR and CNR as well as better CSF suppression at 7 T. Another possible explanation, applicable to both findings, could be that for smaller lesions we are currently at the edge of what can be visualised with the used vessel wall imaging sequences. For these small lesions, scan parameter differences between both field strengths (e.g. slice thickness, voxel sizes) as well as field inhomogeneities may become more important [25]. Consequently, both SNR and CNR may become too low to reliably differentiate lesions from noise, resulting in more subjective assessment. Also, there might be a higher risk of misinterpreting vessel wall irregularities caused by other phenomena like slow flow directly alongside the artery mimicking a lesion, or incomplete CSF suppression [11, 26, 27]. In addition, we can contemplate whether these wall thickenings might actually reflect normal thickness variation throughout the intracranial arterial vasculature. Although two recent post-mortem studies have shown that ultrahigh-resolution 7-T MRI can identify atherosclerotic plaques in intracranial arteries [14, 28], validation of in vivo MRI results with histology has not yet been performed. Therefore, we do not really know to what extent all identified vessel wall lesions in this study are true atherosclerotic lesions, especially when these lesions are seen on one field strength only. This makes it difficult to determine which field strength shows vessel wall lesions best.

One striking secondary result is that in the elderly asymptomatic population included in this study, a substantial amount of intracranial vessel-wall lesions have been found. Most studies have attempted to target symptomatic intracranial atherosclerosis; until now, only transcranial Doppler has been used to assess the presence of intracranial atherosclerosis in an asymptomatic population [29], but this technique solely provides information about the presence of stenotic lesions, i.e. advanced atherosclerotic lesions. Previous studies in a symptomatic patient population with ischaemic stroke/TIA imaged with the same 7-T MRI sequence found 84 % of patients had on average three vessel-wall lesions in the circle of Willis arteries, and 100 % of patients had on average four lesions if the vertebral arteries were also (mostly) taken into account [19, 21]. These results show a striking similarity with our current results of 100 % of subjects with on average five vessel-wall lesions at 7 T; however, our study population consists of asymptomatic healthy elderly volunteers instead of patients with symptomatic cerebrovascular disease.

Another striking result is that in our elderly asymptomatic population, many of the intracranial vessel-wall lesions showed enhancement after contrast administration, predominantly at 7 T. Shortening of the T_1_ relaxation time caused by the injected contrast agent is suggested to be reduced at higher field strengths [30]. However, the increased SNR at 7 T may theoretically make the sequence more sensitive for smaller amounts of contrast agent, e.g. when present in a vessel wall lesion. Also, the 7-T sequence used in this study might be stronger T_1_-weighted than the 3-T sequence, making the 7-T sequence more sensitive for visualising enhancement. Another possible explanation might be that the time between contrast administration and acquisition of the postcontrast vessel-wall scan was not exactly identical during both MRI examinations [8] (approximately 4 min shorter at 3 T). Contrast enhancement of intracranial atherosclerotic plaques has been associated with acute ischaemic stroke [31–33] and could be a marker for plaque inflammation or neovascularisation [34], and even potentially of intracranial plaque instability and stroke risk [33]. Our results, however, show that enhancing lesions are not always associated with (acute) ischaemic stroke or plaque inflammation.

The results from this study shed new light on the clinical application of intracranial vessel wall imaging, and raise important issues that need to be considered. Currently, differentiation between atherosclerotic lesions and normal vessel-wall thickness variations resp. (CSF) flow artefacts alongside the artery walls is difficult. Although much is known already about in vivo vessel-wall (intima-media) thickness of extracranial arteries [35], limited information is available on (normal) vessel-wall thickness variations of the arteries of the circle of Willis. The current study shows vessel-wall lesion burden of the asymptomatic population was comparable to that of symptomatic patients in previous studies. Apart from being vessel-wall lesions, these wall thickenings could also be a reflection of ‘normal’ variation in vessel wall thickening throughout the intracranial arterial vasculature. Therefore, the clinical relevance of these vessel-wall lesions might be limited. This may be especially true for enhancing lesions that, based on our current study, are apparently not always associated with ischaemic stroke or plaque inflammation, as has thus far been assumed. Therefore, future studies comparing both symptomatic and asymptomatic patients should be performed, to gain more insight into differences of vessel-wall lesion burden/thickness variations, as well as contrast enhancement of the vessel wall.

The current study uses a 7-T vessel wall sequence with a relatively low spatial resolution compared with most published (and our own) 3-T sequence(s). A disadvantage of using an inversion recovery pulse (for optimal CSF suppression) is that it increases acquisition time. Therefore, the acquired spatial resolution of the 7-T sequence used in our study is currently restricted to 0.8 × 0.8 × 0.8 mm^3^. As a consequence, some of the scored vessel wall lesions will be smaller than the true voxel size of this 7-T sequence (although this is also true for most used 3-T sequences), since the intracranial vessel wall is typically very thin. However, a previous study by Kleinloog et al. [36], in which the same 7-T vessel-wall sequence was used as in the current study, showed that signal intensity variation of the vessel wall reflects thickness variation. Therefore, it is possible to infer thickness variations from the 7-T vessel-wall images even for vessel walls that are thinner than the acquired voxel size. Also, the advantage of high image contrast between vessel wall (lesions) and surrounding tissue at 7 T, due to the applied inversion recovery pulse, could compensate for, or be even more important than, spatial resolution for visualising vessel wall lesions. Although other research groups have reported vessel wall sequences with higher resolutions, they achieved less optimal CSF suppression [10, 11, 37, 38]. Future work should be done to evaluate the relative importance of resolution versus contrast and CSF suppression.

This study has several limitations. First, the MRI sequences used at both field strengths were not exactly the same: the main differences were the higher in-plane spatial resolution of the 3-T sequence and higher through-plane (slice thickness) spatial resolution of the 7-T sequence, and the lack of an inversion pulse for CSF suppression at 3 T. However, to achieve a fair comparison between both field strengths, we found using optimised imaging sequences for each field strength the best option. Second, the receive coils used in this study were different (eight-channel head coil at 3 T versus 32-channel head coil at 7 T). Third, in this study all minor vessel wall thickness variations were scored as vessel wall lesions. As discussed earlier, especially for small vessel wall lesions, we cannot be certain that these are all actual vessel wall lesions; for translation of the vessel wall imaging findings to clinical practice, as well as usability in future studies, validation with a “gold standard” (histology) is a prerequisite. Fourth, blinding the raters for field strength was not possible due to the inherent slightly different image contrast between 3 T and 7 T; this might have induced a bias in image rating. Fifth, the sample size of this study was relatively small. However, this is the first study to provide a comparison of 3-T and 7-T intracranial vessel-wall imaging, as well as vessel-wall imaging in an asymptomatic elderly population with the administration of a contrast agent to assess enhancement of the vessel wall. Lastly, although 7-T MRI is still less available compared with the lower field strength MR systems, the number of installed 7-T MR systems has increased in recent years [39]. Together with the tremendous growth of technical developments at 7-T MRI over the last decade to overcome difficulties of imaging at higher field strength, its use may become more broadly available in the near future.

In summary, in this comparative study of a dedicated 3-T VIRTA and 7-T MPIR-TSE intracranial vessel-wall sequence in asymptomatic individuals, 7-T vessel-wall imaging appears to have the highest potential to identify the total burden of vessel wall lesions in the arteries of the circle of Willis and its branches. However, a direct comparison between symptomatic and asymptomatic patients is warranted to elucidate the potential for clinical application of intracranial vessel-wall imaging.

## Electronic supplementary material

Below is the link to the electronic supplementary material.

ESM 1 (DOCX 24 kb)
