# Supplementary material for: High-resolution intracranial vessel wall MRI in an elderly asymptomatic population: comparison of 3T and 7T
Source: Eur Radiol. 2016 Jul 7;27(4):1585–95. doi: 10.1007/s00330-016-4483-3 (PMC5334422; doi:10.1007/s00330-016-4483-3)
Supplement: Supplementary file 1 — (DOCX 24 kb) [file 330_2016_4483_MOESM1_ESM.docx]

**SUPPLEMENTAL MATERIAL**

**Supplementary – Vessel wall lesion characteristics**

In addition to contrast enhancement, intracranial vessel wall lesions were also assessed for specific lesion characteristics, according to the methods previously described.[[1-3](#_ENREF_1)] Vessel wall lesion characteristics included: (1) configuration: eccentric (thickening clearly limited to one side of the vessel wall or if the thickest part was more than twice the thinnest part) or concentric (uniform and circumferential thickening); and (2) thickening pattern: focal or diffuse (thickening extending over a longer distance). The characteristics of vessel wall lesions were scored on the precontrast vessel wall images. Interrater agreement of vessel wall lesion characteristic (configuration and thickening pattern) assessment was calculated using Cohen’s kappa, and showed a good to excellent interrater agreement for assessment of configuration (kappa 0.81 for 3T, and 0.82 for 7T) and thickening pattern (kappa 0.73 for 3T, and 0.78 for 7T) of each vessel wall lesion scored by both raters. However, in the side-by-side comparison, a poor agreement was found between 3T and 7T for assessment of configuration (kappa 0.06) and thickening pattern (kappa 0.04) of the corresponding vessel wall lesions. Therefore, any possible correlation between lesion characteristics and field strength could not be assessed. This might be explained by the same reason previously mentioned in the Discussion section. For the small lesions identified in this study we are currently working at the edge of what can be visualized with these intracranial vessel wall sequences. Therefore, for these lesions both the SNR and CNR may become too low to reliably differentiate them from noise in the image, resulting in more subjective identification of lesions as well as their characteristics.

**Supplementary References**

1. van der Kolk AG, Zwanenburg JJ, Brundel M, et al. (2011) Intracranial vessel wall imaging at 7.0-T MRI. Stroke 42: 2478-2484.

2. Swartz RH, Bhuta SS, Farb RI, et al. (2009) Intracranial arterial wall imaging using high-resolution 3-tesla contrast-enhanced MRI. Neurology 72: 627-634.

3. Dieleman N, van der Kolk AG, van Veluw SJ, et al. (2014) Patterns of intracranial vessel wall changes in relation to ischemic infarcts. Neurology 83: 1316-1320.

**Supplemental Table I.** Baseline characteristics of all 21 subjects.

| Characteristic, n(%) | | |
| --- | --- | --- |
| Gender, male |  | 12 (57.1%) |
| Age, years (mean ± sd) |  | 65.9 ± 5.0 |
| BMI, kg/m^2^ (mean ± sd) |  | 24.8 ± 3.3 |
| Smoking | *current* | 1 (4.8%) |
|  | *former* | 10 (47.6%) |
|  | *never* | 10 (47.6%) |
| Blood pressure, mm Hg (mean ± sd) | *systolic* | 141 ± 24 |
|  | *diastolic* | 80 ± 9 |
| History of hypertension |  | 8 (38.1%) |
| Hypercholesterolemia |  | 6 (28.6%) |
| Diabetes mellitus |  | 1 (4.8%) |
| History of cardiovascular disease |  | 1 (4.8%) |
| Family history vascular disease < 60 y |  | 5 (23.8%) |

*BMI: body mass index*
